# Report of a Case of Intussusception of the Bowel

**Published:** 1880-06

**Authors:** N. B. Kennedy

**Affiliations:** Hillsboro, Texas


### REPORT OF A CASE OF INTUSSUSCEPTION OF THE BOWEL.

Read before the Hill County Medical and Surgical Association,

By N. B. KENNEDY, M.D., Hillsboro, Texas.

*Mr. President and Gentlemen:* I wish to offer to-day for your consideration a few remarks on intussusception of the bowel, a condition most generally proving fatal, but fortunately of not very frequent occurrence. Intussusception is understood to be an introduction of one portion of intestine by inversion into the portion either above or below it, thus frequently closing the cavity of the intestine entirely. The invagination generally occurs from above downwards, but not invariably so. The invagination may be found from less than one inch in extent to a foot or more. It may occur in more places than one at the same time, and along any portion of the intestinal tract. The diagnosis is at first always uncertain. It may, however, be suspected when symptoms of obstruction come on suddenly, without any previous disturbance, as an attack of colic, or the appearance of a tumor in the colon when none previously existed. The patient often becomes very restless at the beginning; extreme jactitation, with great desire to assume an easy posture, but with inability to do so; uneasiness in the bowel, with frequent desire to evacuate. Cathartics and enemata generally fail to produce any discharge, except the inodorous water injected. The surface becomes cool and clammy; the tongue heavily coated with a yellowish fur; great anxiety of countenance; hurried respiration; pulse small and quick; the abdomen becomes swollen and tender on pressure; nausea and vomiting now take place; stercoraceous matter being ejected from the stomach. And finally the extremities become cold, the pulse at the wrist so exceedingly small and feeble, as to be almost imperceptible; the countenance haggard, delirium taking place, and finally death mercifully closes the scene.

It need hardly be mentioned that the prognosis is almost always unfavorable.

I was called May 29th, 1880, at about 2 o'clock P.M., to see little Effie V——, a beautiful little girl, with bright sparkling eyes and glossy curling hair, of four years. I found her with an accelerated pulse, hurried breathing and some elevation of temperature, and her tongue coated with a yellowish fur. She was complaining of some pain about the region of the umbilicus, and was extremely restless, tossing about from side to side of the bed; occasionally rising and attempting to go out of doors. Her bowels had not acted for twenty-four hours, and she had great thirst. I gave her the following prescription:

℞.—Extract colocynth, comp.; pulv. rhei.; hydragyri chloride mitis, aa. gr., xx. Divide into four powders.

Sig.—One powder every two hours until the bowels act.

I gave her also a febrifuge, and ordered quinine as soon as the fever should subside. Having several other urgent calls to make, I left, telling the family I would return as soon as possible.

At 8 o'clock P.M., of the same day, was called in great haste to see my little patient. I found her, on arrival, bathed in a cold, clammy sweat; extremities cold; great restlessness and anxiety; abdomen distended and severe spasmodic pain; tenderness on pressure; pulse quick; tongue deeply furred.

On inquiry, I learned the patient had taken and retained all the powders which I had prescribed, but they had failed to act on her bowels. I ordered a hot hip bath, placed the child in it for ten minutes, and had her thoroughly rubbed dry with a coarse towel; applied hot turpentine fomentations to the abdomen; gave large enemata of warm soap suds; warm water, salt and castor oil, with no amelioration of the symptoms whatever. I then introduced an enema pipe five inches long into the rectum, and gently pushing it up, soon came in contact with what I supposed the obstruction, which I diagnosed invagination of the colon. I threw up a gallon or two of warm soap suds against the obstruction; a large quantity of warm water and salt, but all to no effect. Finally, I tried to pass the tube of the syringe beyond the obstruction, but could not.

During this treatment, I gave opium and stimulants freely, but to no appreciable effect. By this time the unfavorable symptoms which had existed from the first, had become greatly augmented, together with extreme nausea and stercoraceous vomiting. Nervous prostration had come on so rapidly and was so great that I could not for a moment entertain the idea of performing colotomy. Having twice performed it for intussusception of the bowel, and on both occasions only hastening the death of my patients, I here solemnly protest against said operation.

It is true if the diagnosis could be made out with unerring certainty at once and the operation immediately performed, there would be a remote contingency of prolonging life, but even in such cases I think it a surgical procedure of doubtful propriety.

The abdomen of the child had now become very much distended and tympanitic; respiration oppressed; face bathed in cold sweat; skin pale and clammy; extremities cold; mind wandering; pulse imperceptible and the countenance haggard.

And finally, this beautiful little girl, the idol of her fond parents, and the pet of the household, succumed to this usually fatal condition of strictured bowels.

